# Mycosis fungoides diagnosed after exposure to risankizumab for psoriasis

**DOI:** 10.1016/j.jdcr.2023.08.049

**Published:** 2023-09-27

**Authors:** Lauren M. Fahmy, Celine M. Schreidah, Brigit A. Lapolla, Cynthia M. Magro, Larisa J. Geskin

**Affiliations:** a Columbia University Vagelos College of Physicians and Surgeons, New York, New York; b Department of Dermatology, Columbia University Irving Medical Center, New York, New York; c Department of Pathology and Laboratory Medicine, Division of Dermatopathology, Weill Cornell Medicine, New York, New York

**Keywords:** biologic, cytokine blocker, interleukin-23, mycosis fungoides, psoriasis, risankizumab

## Introduction

Although biologics are increasingly used to treat inflammatory dermatoses, concerns have been raised about their long-term safety. There is an established link between tumor necrosis factor-α inhibitors and cutaneous T-cell lymphoma (CTCL),^1^ and several reports describe worsening CTCL after exposure to other cytokine blockers.^1^^,^^2^ We present a patient who was diagnosed with mycosis fungoides (MF) after treatment with risankizumab, an IL-23 inhibitor, for psoriasis.

## Case report

A 54-year-old man presented to dermatology for the evaluation of a 4-month history of worsening rash. He reported a history of psoriasis since childhood that was clinically diagnosed. His psoriasis was intermittent and well controlled on low-potency topical steroids for many years. One year prior, he noticed dystrophic nail changes. Three months after noticing these changes, he began on risankizumab 150-mg injections for nail psoriasis. Within 4 months, he had marked improvement in his nail symptoms but developed new, faintly erythematous patches on his trunk, back, and thighs that looked different in nature than his previous psoriasis lesions. Toenail clippings and a skin biopsy were obtained. Skin biopsy revealed an atypical CD4-dominant epitheliotropic lymphocytic infiltrate, which was suspicious for evolving T-cell dyscrasia. Nail plate clippings showed morphologic evidence of psoriasis without onychomycosis. He was referred to the Columbia Comprehensive Cutaneous Oncology Center for further evaluation.

**Fig 1:**
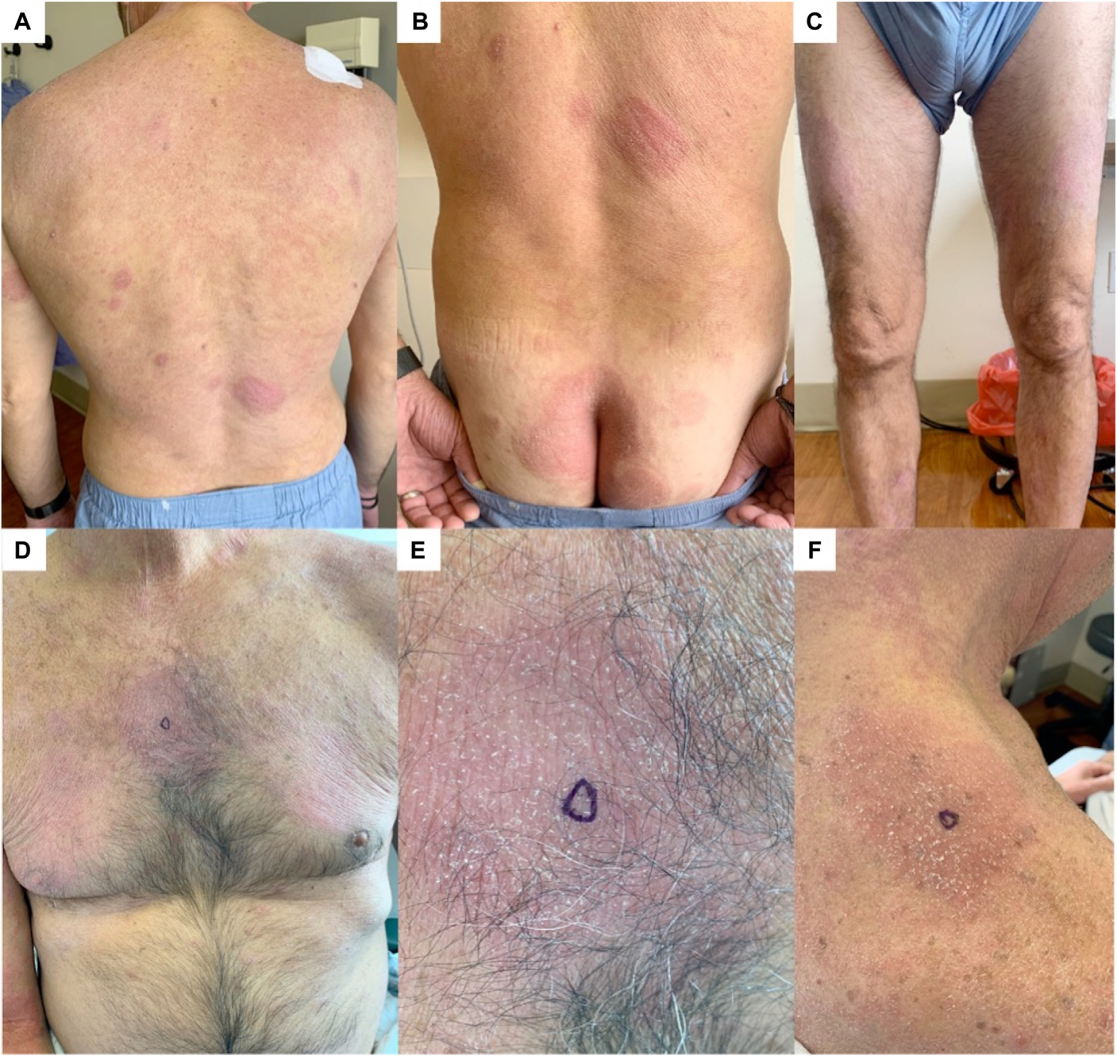
Mycosis fungoides arising on a background of follicular psoriasis. **A,B,** Well-demarcated, erythematous scaly plaques are present on the back and gluteal cleft. **C,** Faint pink patches spanning up to 10 cm in diameter are seen on the anterior aspect of the thighs. **D,** Faint pink patches are distributed across the chest. **E,F,** Follicular prominence can be appreciated in some patches on the trunk.

**Fig 2:**
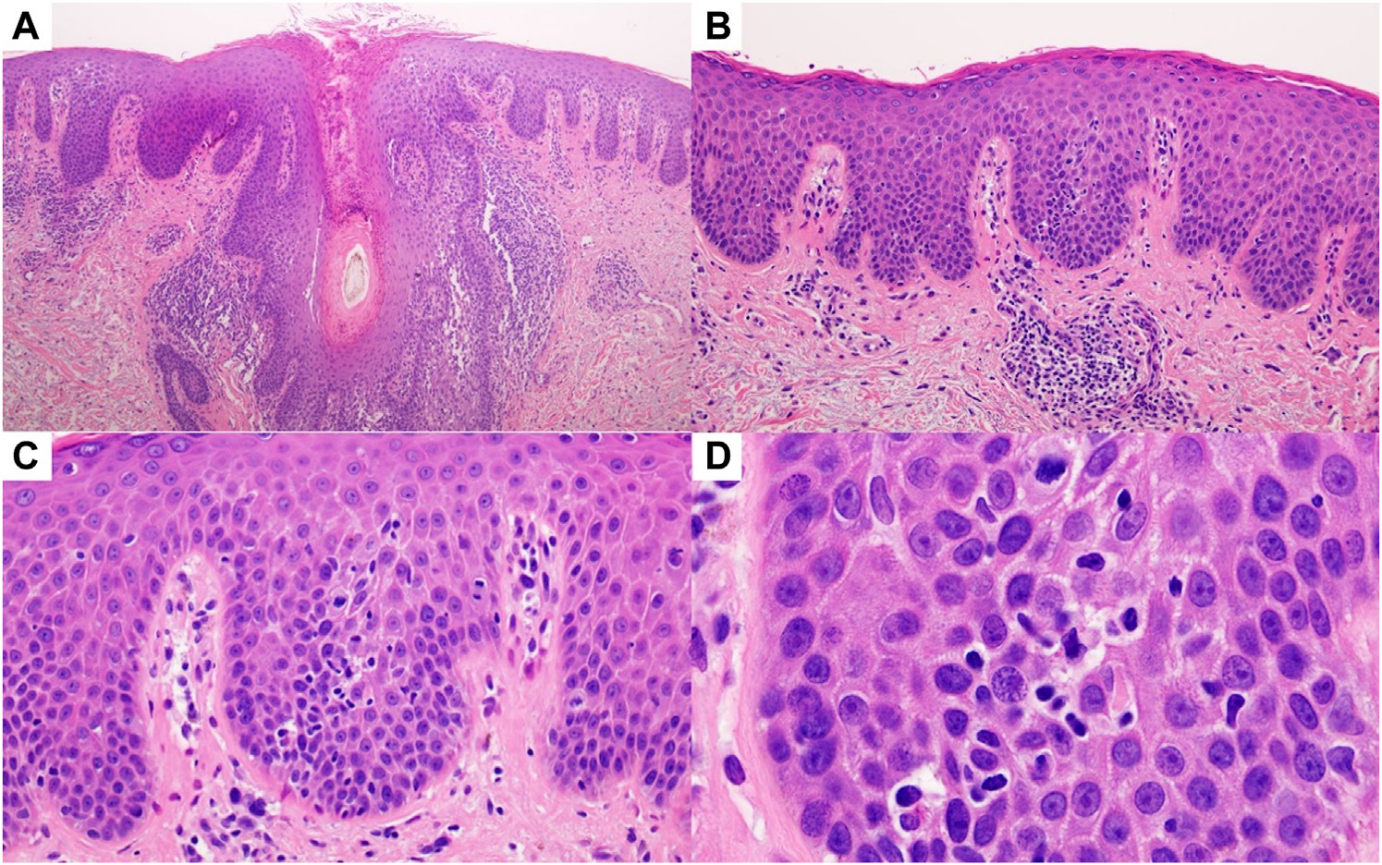
**A,** A low-power view of the skin biopsy demonstrates a psoriasiform epidermal hyperplasia and follicular plugging by keratin. The outer root sheath epithelium is infiltrated by neutrophils, and the follicle is surrounded and permeated by lymphocytes. Perivascular collections of lymphocytes are seen. (Hematoxylin-eosin stain; original magnification: 40×.) **B,** There is an epitheliotropic lymphocytic infiltrate, with lymphocytes present within the basal layer with some degree of upward migration to the spinous layer. (Hematoxylin-eosin stain; original magnification: 100×.) **C,** Higher magnification highlights the relatively passive pattern of lymphocyte migration into the epidermis. The dermal papillae are somewhat hypervascular, whereby the capillaries are dilated and focally juxtaposed to the basal layer of the epidermis. (Hematoxylin-eosin stain; original magnification: 200×.) (**D**) Higher power oil examination highlights the atypia of the epidermotropic lymphocytes. (Hematoxylin-eosin stain; original magnification: 400×.)

**Fig 3:**
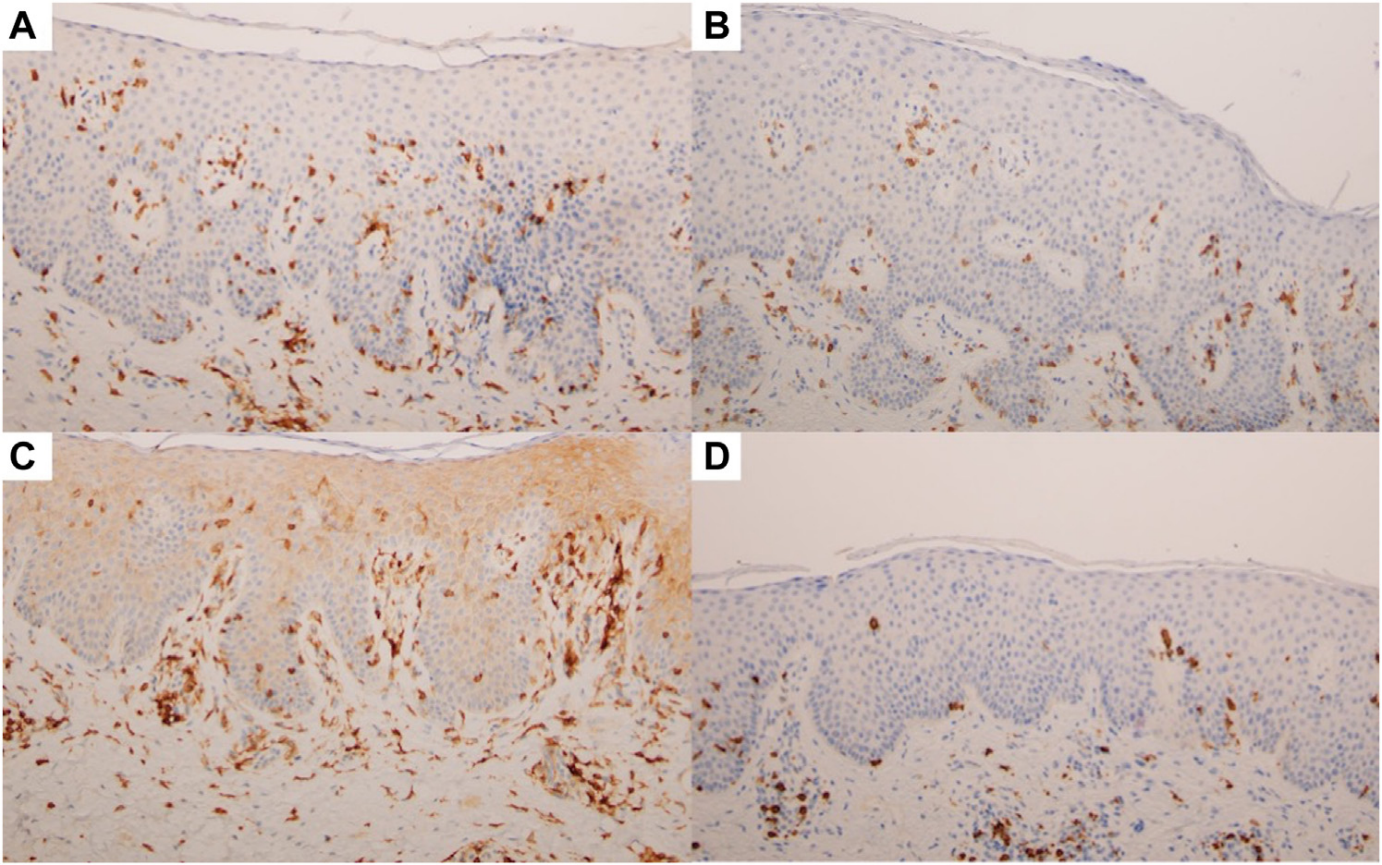
**A,** CD3 stain highlights the extent of the interadnexal epitheliotropic lymphocytic infiltrate (diaminobenzidine, CD3; original magnification: ×200). **B,** CD7 stain discloses a significant loss of CD7 (diaminobenzidine; CD7; original magnification: ×200). **C,D,** CD4 and CD8 stain, respectively, reveal a skewed intraepithelial CD4:CD8 ratio approaching 10:1. (**C** and **D,** CD4 and CD8; diaminobenzidine; original magnifications: **C,** ×200; **D,** ×100.)

On physical examination, he had erythematous, scaly, well-demarcated plaques on the face, neck, back, buttocks, arms, and legs. He also had numerous faintly erythematous patches of variable shape scattered across his thighs, abdomen, flank, and back ranging from 1 to 5 cm in diameter, some with follicular prominence (Fig 1). He had onychodystrophy of all toenails and fingernails with thickened hyperkeratotic subungual debris. He had no lymphadenopathy. Risankizumab was discontinued, and he initially responded well to significant recreational UV exposure but relapsed 5 months later during the winter when he had minimal sun exposure. Additional punch biopsies were obtained and showed classic features of psoriasis with follicular involvement. The latter was characterized by striking follicular hyperkeratosis with parakeratosis, entrapment of neutrophils, and dilated dermal papillary capillaries (Fig 2, *A*). However, the biopsies also showed evidence of an atypical lymphoid process characterized by passive infiltration of the epidermis and hair follicle by small- to intermediate-sized atypical lymphocytes exhibiting nuclear contour irregularity, including cells with a cerebriform appearance (Fig 2, *B* and *D*). An aberrant phenotype was revealed by the extent of loss of CD7 relative to CD3 (Fig 3, *A* and *B*), and there was a skewed CD4:CD8 ratio approaching 10:1 (Fig 3, *C* and *D*). The findings were consistent with an epidermotropic T-cell dyscrasia arising on a background of follicular psoriasis. Peripheral blood flow cytometry was unremarkable. A positron emission tomography/computed tomography scan revealed bilateral inguinal lymphadenopathy (maximum standardized uptake value 3.4), which was thought to be attributable to chronic reactive changes. Taken together, the clinical, pathologic, and immunohistochemical findings were consistent with early patch-stage MF (stage IA, T1bNxM0B0) based on the diagnostic algorithm proposed by the International Society for Cutaneous Lymphomas.^3^ He began acitretin 50 mg daily and narrow-band UV-B therapy 3 times weekly, which led to moderate clinical improvement.

## Discussion

There are now numerous reports of new CTCL diagnoses after biologic use. Among the 4 currently available biologics that block IL-23, only ustekinumab and guselkumab have been associated with new onset of CTCL. Several patients with psoriasis have been reported to develop MF after treatment with ustekinumab, an antagonist of IL-12 and IL-23. There is one report of a patient developing MF after receiving guselkumab, an IL-23 inhibitor; however, this patient also received dupilumab and adalimumab during her treatment course, making it difficult to discern which medication(s) were linked to her subsequent MF diagnosis.^4^ To our knowledge, this is the first report of MF developing after exposure to risankizumab, a selective IL-23 blocker, suggesting that modulation of IL-23 alone in some patients may be sufficient to unmask or trigger CTCL.

To determine whether CTCL has been reported as an adverse event associated with risankizumab in other patients, we queried the Food and Drug Administration Adverse Event Reporting System public dashboard, a database containing adverse event reports submitted by health care professionals, consumers, and manufacturers, on July 24, 2023. We identified 14 reports of CTCL associated with risankizumab, including 8 cases reported in 2021 and 6 cases reported in 2022. These data should be interpreted with caution because there are several limitations of the Food and Drug Administration Adverse Event Reporting System database, including the possibility for incomplete and duplicate reports, unverified data, and limited information about the patients who experienced the reported adverse events (eg*,* no information about other medications patients were taking at the time of the adverse events). Nonetheless, these results suggest that there may be additional cases of MF diagnosed after exposure to risankizumab.

The patient’s history of psoriasis since childhood, his biopsy showing typical features of psoriasis with follicular involvement, including neutrophils infiltrating the outer root sheath epithelium of the hair follicle and dilated dermal papillary capillaries, and his biopsy-confirmed nail psoriasis suggest that the patient had a true background of psoriasis. Because he did not have histologic evaluation of his rash prior to starting risankizumab, it remains unclear whether the drug induced the development of atypical cells or whether he already had an atypical lymphoid process that was accelerated by the drug. Regardless, the patient’s rapidly worsening rash after initiating risankizumab, in the absence of other medications or identifiable triggers, suggests a link. Both MF and a drug-associated reversible T-cell dyscrasia were considered in the differential diagnosis; however, MF was favored based on the duration of the patient’s cutaneous lesions beyond 6 months after risankizumab cessation.^5^

**Fig 4:**
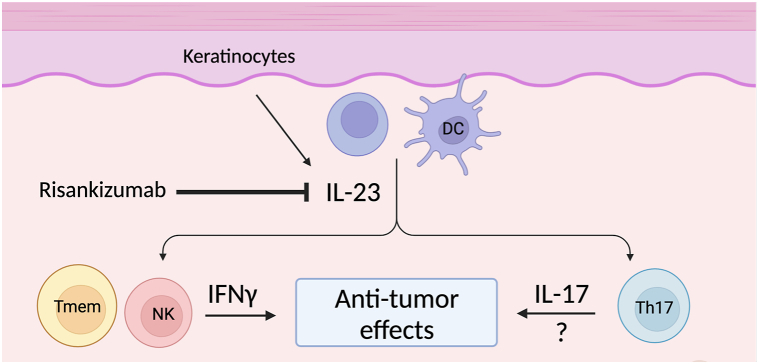
Schematic of the hypothesized mechanisms by which risankizumab may unmask mycosis fungoides. Keratinocytes, dermal lymphocytes, and antigen-presenting cells, such as dendritic cells (DCs) produce interleukin (IL) 23. IL-23 stimulates memory T cells (Tmem) and natural killer (NK) cells to produce interferon gamma, a cytokine that has various antitumor effects in cutaneous T-cell lymphoma (CTCL), including activation of cytotoxic T cells, priming of DCs, and reduction of tumor cell proliferation. IL-23 also promotes the differentiation of Th17 cells, which produce large quantities of IL-17. IL-17 may have anticarcinogenic properties in some patients with CTCL, although the mechanism by which this occurs is unclear. In predisposed patients, risankizumab may unmask mycosis fungoides by reducing downstream levels of IFN-γ and IL-17, attenuating their antitumor effects. These hypotheses have not been confirmed and require additional investigation. Created with BioRender.com.

Currently, the role of IL-23 in CTCL pathogenesis remains unclear. IL-23 is elevated in several inflammatory and autoimmune diseases, including psoriasis, and promotes the expansion of Th17 cells, which produce IL-17, IL6, and tumor necrosis factor-α. Doherty et al^6^ demonstrated that IL-23 is upregulated in epidermal keratinocytes and in dermal lymphocytes in MF/Sézary syndrome lesions of all stages compared with normal skin but absent or weakly expressed in atypical lymphocytes in advanced lesions. Considering that IL-23 can induce interferon (IFN) gamma production by memory T cells,^7^ and IFN-γ has antiproliferative effects in CTCL,^8^ suppression of IFN-γ by IL-23 blockade may fuel the expansion of malignant T cells. One may also consider whether the effects of IL-23 inhibition are because of a downstream reduction in IL-17 given the dominant role of IL-23 in promoting Th17 cells. The data regarding the role of IL-17 in CTCL are mixed, with some studies demonstrating that IL-17 expression is associated with disease progression^9^ and others suggesting the opposite.^10^ Nonetheless, there are several reported cases of IL-17 inhibitors exacerbating CTCL, indicating that IL-17 may have antitumor effects in at least a subset of patients with CTCL.^2^ Therefore, modulation of IFN-γ and/or IL-17 provide possible mechanistic links between IL-23 inhibitors and CTCL development (Fig 4). These hypotheses require further study because there may be other mechanisms at play.

Although additional investigation is needed to understand the biology of IL-23 in CTCL, we propose that IL-23 blockade may contribute to T-cell dysregulation in predisposed individuals. Close monitoring of patients with psoriasis on risankizumab is warranted, and CTCL should be considered in patients who worsen or develop new rashes on this biologic drug.

## Conflicts of interest

Dr Geskin has served as an investigator for and/or received research support from Helsinn Group, J&J, Mallinckrodt, Kyowa Kirin, Soligenix, Innate, Merck, BMS, and Stratpharma; on the speakers’ bureau for Helsinn Group and J&J; and on the scientific advisory board for Helsinn Group, J&J, Mallinckrodt, Sanofi, Regeneron, and Kyowa Kirin. Authors Fahmy, Schreidah, Lapolla, and Magro have no conflicts of interest to declare.
